# Supplementary material for: Longitudinal intravital imaging of the femoral bone marrow reveals plasticity within marrow vasculature
Source: Nat Commun. 2017 Dec 18;8:2153. doi: 10.1038/s41467-017-01538-9 (PMC5735140; doi:10.1038/s41467-017-01538-9)
Supplement: Supplementary file 1 — Supplementary Information [file 41467_2017_1538_MOESM1_ESM.pdf]

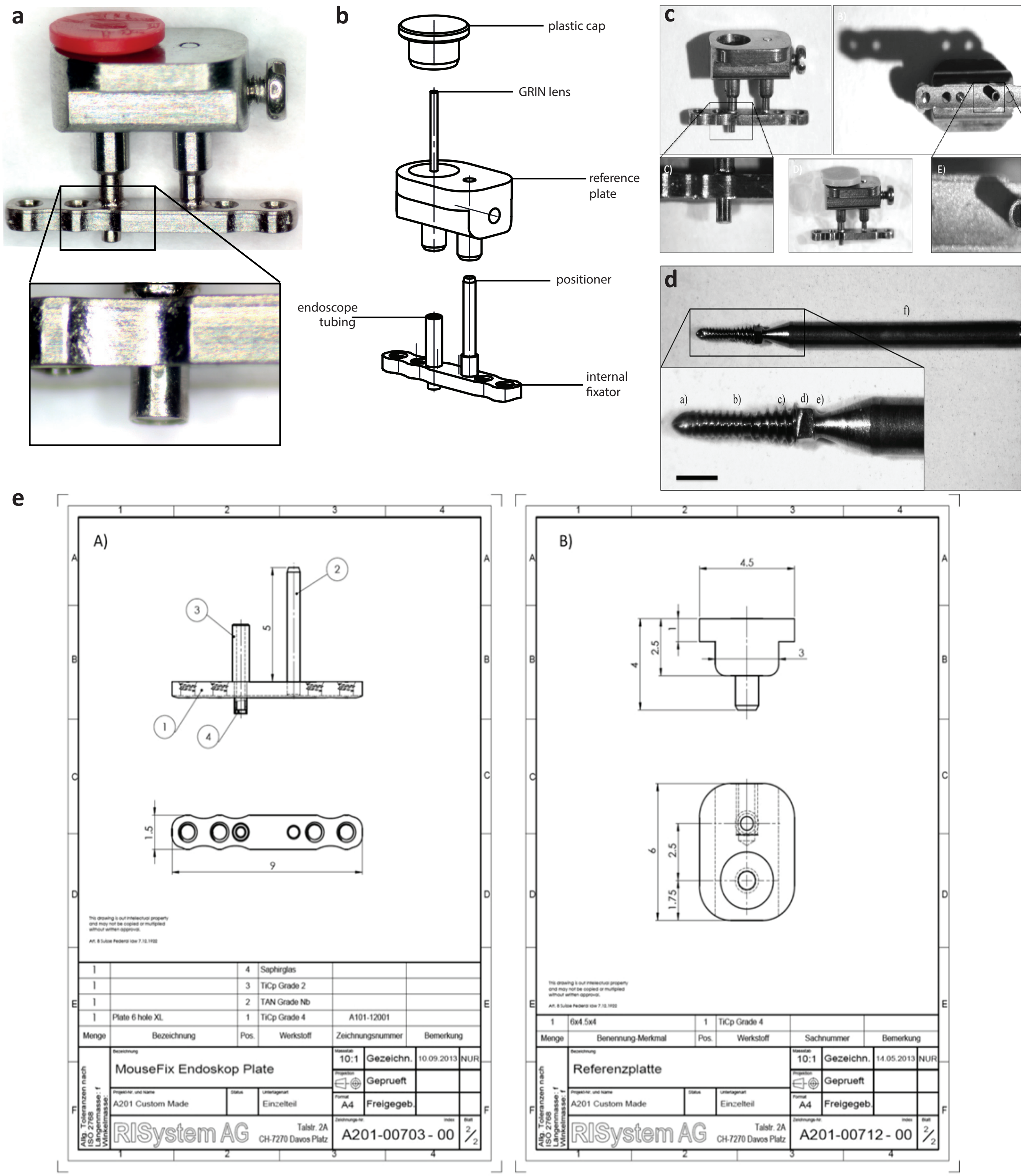

Supplementary Figure 1 | Assembly and technical specifications of the LIMB microendoscope implant. (**a**) The titanium alloy (medical grade) implant consists of two parts that are assembled at the end of the surgery. The extracorporeal reference plate is attached to the positioner and the endoscope tubing and fixed with a screw (M1x2). (**b**) Exploded assembly drawing of the LIMB setup. The plastic cap prevents the endoscope from dust and smaller particles of the cage bedding. The GRIN lens is fit into the endoscope tubing. The endoscope tubing has an inner diameter of 450 µm and an outer diameter of 650 µm. Both, endoscope tubing and positioner, are micro-welded to the internal fixator. (**c**) The sapphire window (d = 500 µm), which seals the tubing at the intramedullary end, is pressed into a recess (right inset). The outer tip ends in the conical recess of the reference plate (left inset) and can be accessed by the microscope objective, when the plastic cap is removed. The reference plate is used for the alignment of the GRIN lens with respect to the microscope objective. It holds the implant and the femur in a stable position under a customized imaging stage (Supplementary Figure 2). (**d**) The bicortical screw (RISystem, Davos, Switzerland) has two different threads b) and c), whereas b) locks into the bone and c) into the fixator plate. d) is the square box wrench for removal of the screw and e) the predetermined breaking point to shear off the shaft f) from the screw at a defined torque. All implanted materials can be autoclaved and are resistant to organic solvents. (**e**) Technical drawings of both parts with relevant dimensioning.

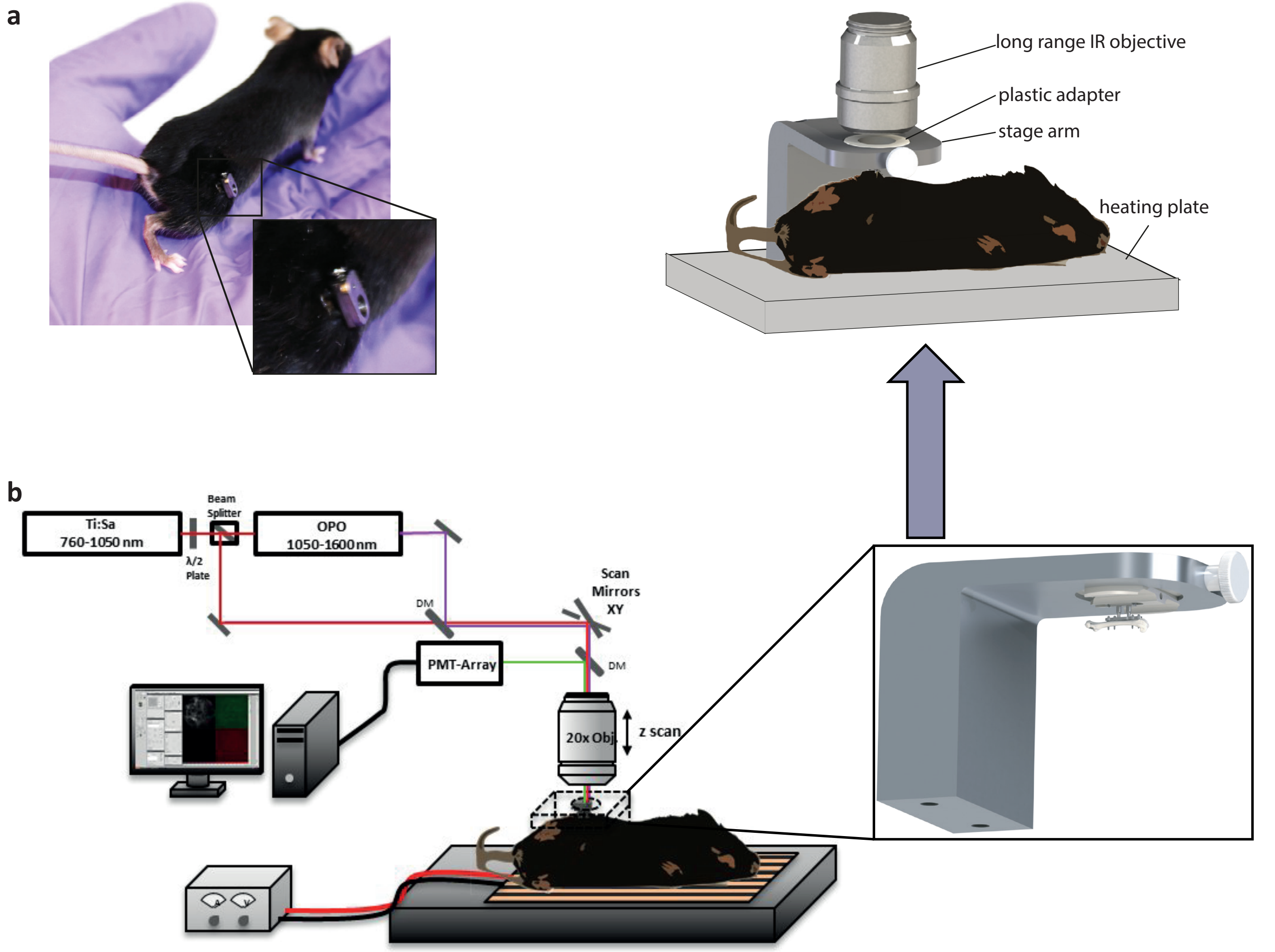

Supplementary Figure 2 | Microscope setup for longitudinal intravital imaging of the bone marrow in mice. (**a**) Photograph of a mouse carrying the LIMB implant 28 days post-surgery. Externally, only the reference plate is visible (inset). (**b**) The mouse is positioned under the two-photon microscope using a customized plastic adapter and stage, so that the GRIN lens is aligned parallel to the optical axis of the microscope objective lens for optimal illumination of the bone marrow tissue. During time lapse image acquisition the animals lay on a controlled heating plate to maintain a constant body temperature.

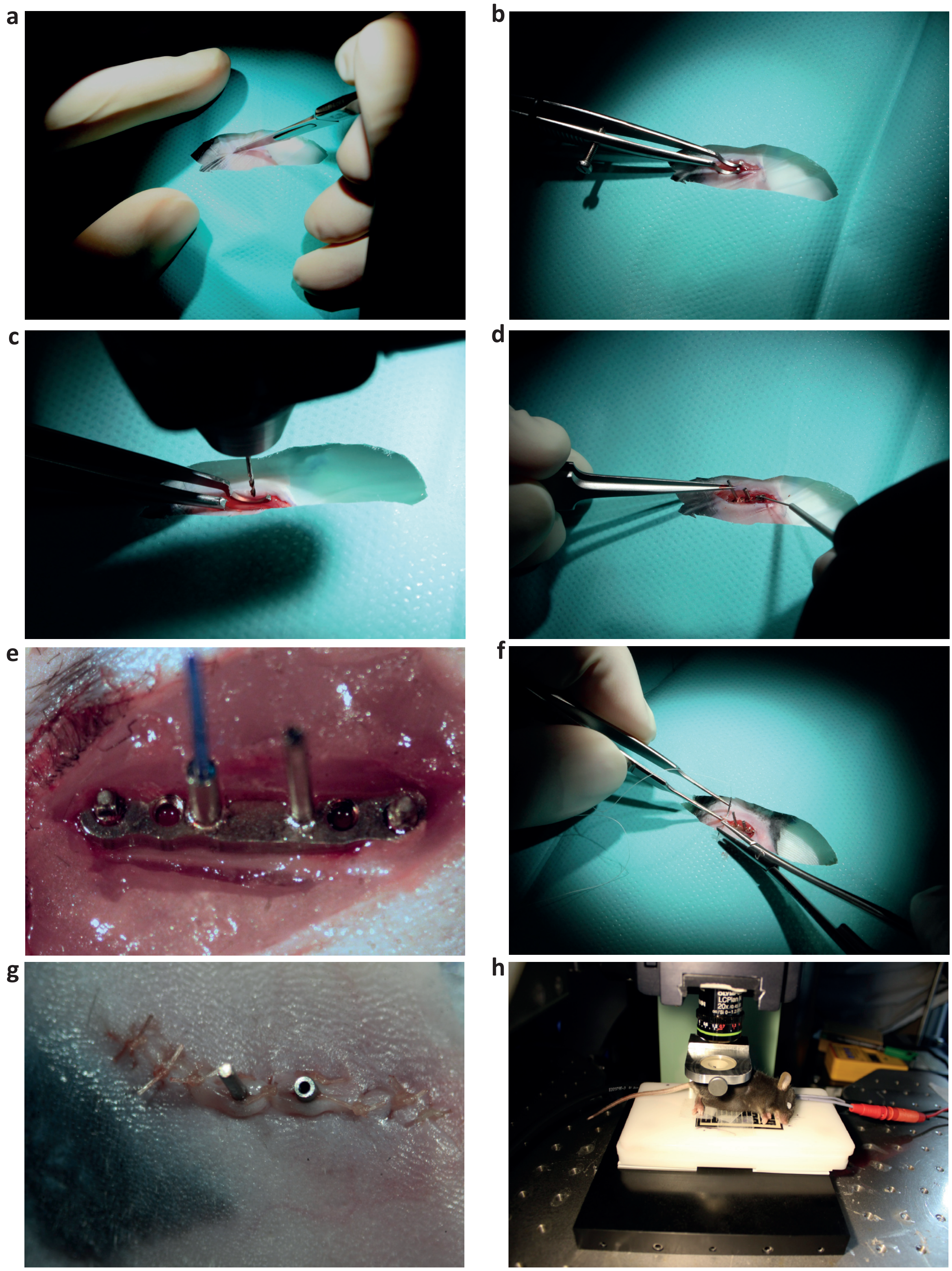

Supplementary Figure 3 | Surgical procedure for implantation of the LIMB-implant. (**a**) A surgical incision is placed into the shaved and disinfected skin between the knee and hip joint, parallel to the femur in order to expose the femoral shaft of the right hind limb. (**b**) A ring forceps is used to fixate the bone. (**c**) For drilling of the pilot hole in the distal half of the diaphysis an electric precision drill and a stand is used. (**d**) The LIMB implant system is placed parallel onto the femur and (**e**) bicortical screws are inserted to complete the fixation. (**f**) The wound was closed with a absorbable surgical thread. In (**g**) the implant after wound closure and before attachment of the reference plate is shown. (**h**) Anesthetized mouse under the two-photon microscope.

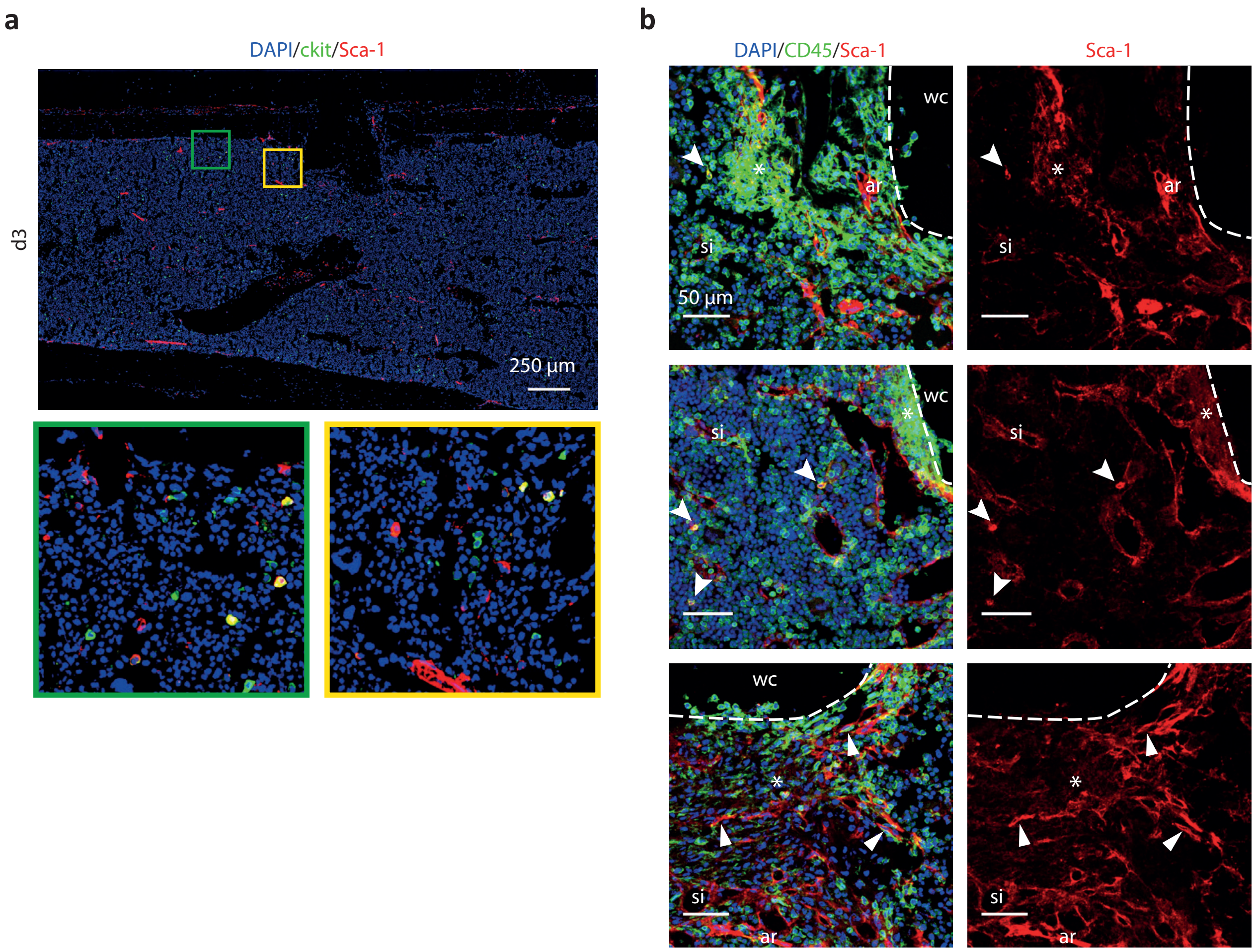

Supplementary Figure 4 | Immunofluorescence analysis early post-surgery. (**a**) Immunofluorescence staining for ckit+sca1+ cells (yellow) shows presence of this HSPC enriched population in endosteal areas and adjacent to the implant window as early as 3 days post-surgery indicating the maintenance of hematopoietic precursor retention in the presence of the LIMB implant (**b**) The tissue adjacent to the window cavity (wc) after removal of the implant displays CD45+ cell (green) accumulations 3 days post-surgery with predominantly rounded nuclei and areas of dim Sca-1 expression (asterisks). Sca-1hi arteriolar endothelial cells with elongated nuclei (triangles) spread into the damaged tissue indicating vascularization. Some cells are Sca1+CD45+ (arrow heads), and might represent hematopoietic precursors. Sinusoids, si; arterioles, ar.

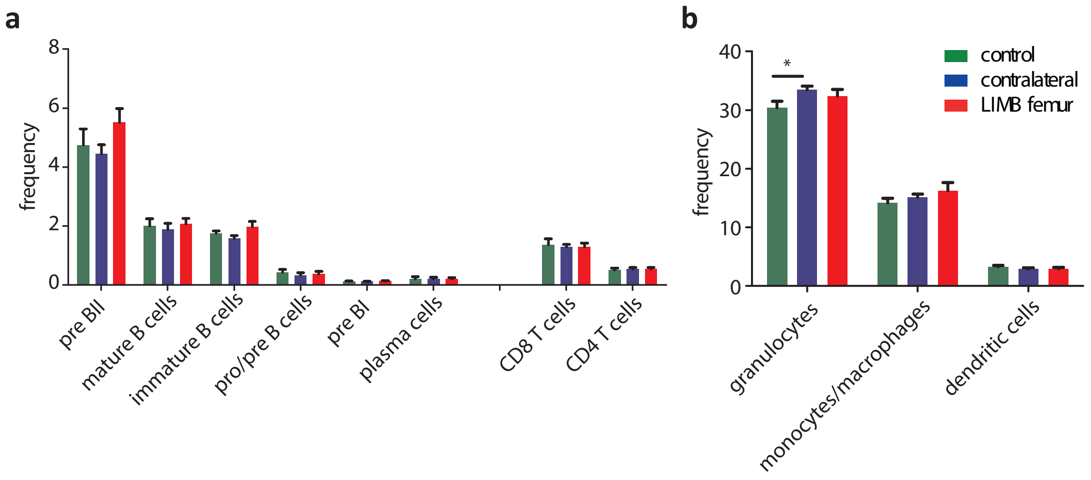

Supplementary Figure 5 | Flow cytometric analysis demonstrates that LIMB has no effect on bone marrow cell populations. Flow cytometry analysis of femurs with the LIMB implant, their contralateral femurs and femurs of control mice. Similar frequencies and cell counts of various leukocyte populations indicate no effect of the presence of the LIMB implant on bone marrow cell composition for (**a**) B and T cell populations and (**b**) innate immune cells. (error bars represent s.e.m., statistical analysis: t-test, * - p = 0.0318, n = 8 LIMB-implanted mice, n = 8 control mice, two independent experiments).

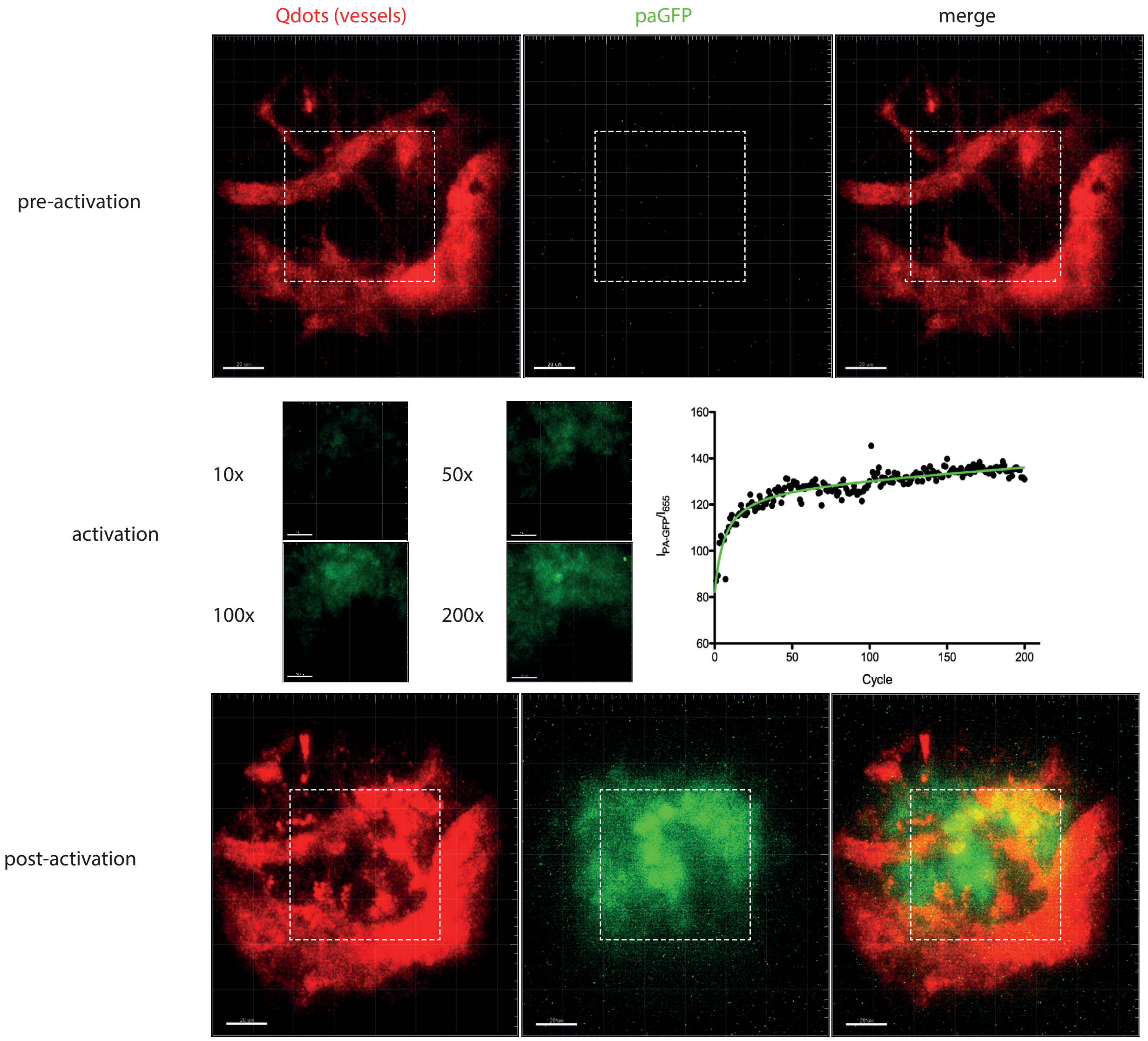

Supplementary Figure 6 | Photoactivation in the bone marrow of paGFP mice using LIMB. Mice ubiquitously expressing paGFP received the LIMB implant and were injected prior to the imaging session with Qdots to label the vasculature. In the course of a photoactivation experiment, first, the pre-activation state of the tissue in the volume-of-view was recorded (upper panel). The vessels were clearly visible but no signal above background was recorded in the paGFP-channel at an excitation wavelength of 940 nm. For photoactivation, the excitation wavelength was tuned to 840 nm and the scanning volume (x, y, z) was reduced from 150x150x30 µm³ to 75x75x30 µm³ (step size 3 µm each) or from 300x300x70 µm³ to 100x100x9 µm³ (step size 3 µm each). During the 200 activation cycles over the time course of 40 min, we recorded the fluorescence signals from the Qdots (vessels) and paGFP as shown in the middle panel. The increase in fluorescence intensity of paGFP ($I_{PA\text{-}GFP}$) normalized to the fluorescence intensity of the vessels (I_{655}) over the number of activation cycles is given in the graph. For multi-photon image acquisition post-activation the excitation wavelength was tuned back to 940 nm and the volume-of-view increased. The same channels were used to record again a 150x150x30 µm³ volume, which clearly shows the activated paGFP inside the cells (lower panel). Scale bar = 20 µm in the upper and lower panel, scale bar = 15 µm in the middle panel.

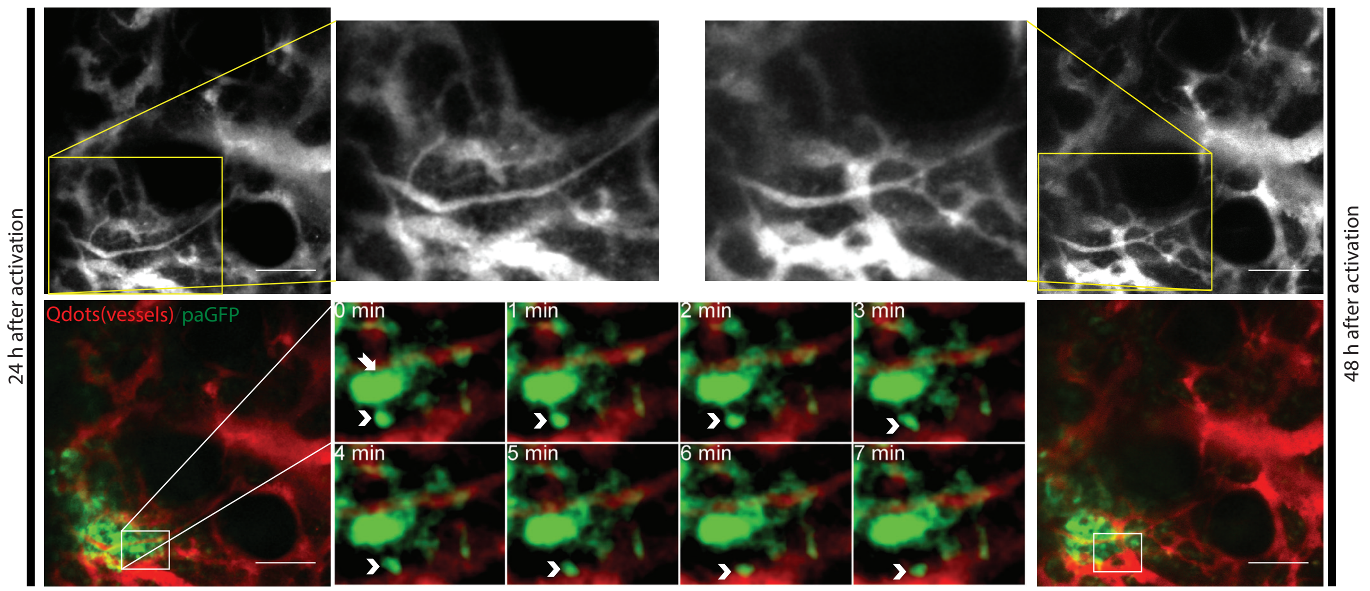

Supplementary Figure 7 | Similar motility patterns of hematopoietic cells in the calvarial bone marrow after photoactivation as compared to the femoral bone marrow. The photoactivation was performed within a volume of a 150x150x9 µm³ region within the 500x500x66 µm³ field of view. The fluorescence images were longitudinally acquired 24 h and 48 h after activation. The upper panel shows the vessel network in the calvarial bone and bone marrow (gray) 24 h after activation and 48 h after activation with enlarged inserts demonstrating changes in the vasculature within one day. The lower panel shows vessels in red and activated paGFP in green. The lower middle panel shows time-lapse 3D images of the inset in the left panel, indicating paGFP fluorescent cells migrate out of the initial photoactivation volume 24 h after photoactivation. They are fluctuating in number and position within the tissue within short periods of time (7 min). Scale bars = 100 µm.

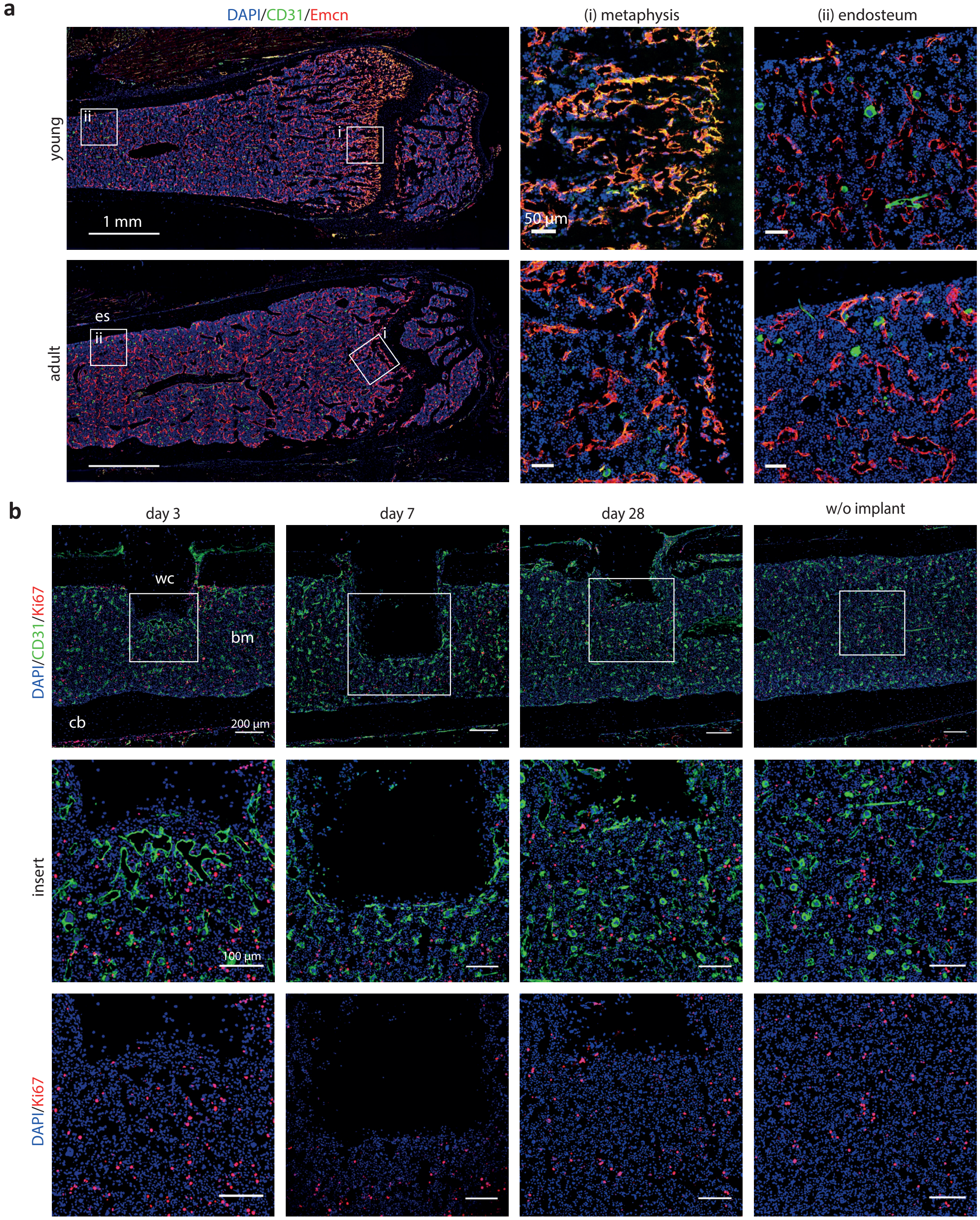

Supplementary Figure 8 | Presence of type H vessels in the bone marrow in different age cohorts and proliferation of bone marrow cells assessed by KI-67 staining. (**a**) Immunofluorescence analysis shows that type H vessels (EmcnhiCD31hi) are more abundant at the femoral growth plate of young mice (4 weeks old) as compared to old mice (18 weeks old). (**b**) Immunofluorescence analysis of nuclear Ki67 hardly stains for endothelial cells. Proliferation was heterogeneously distributed at different times, similar to bones without (w/o) implant.

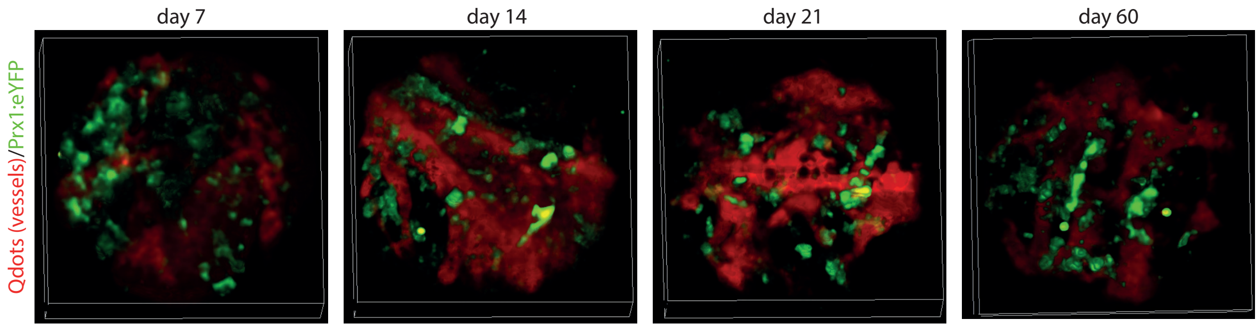

Supplementary Figure 9 | LIMB analysis in Prx1:YFP mice reveals continuous remodeling of the stroma compartment in the mouse femur over months. Prx1:YFP mice, which express eYFP in stromal and endothelial cells, received the LIMB implant and were injected prior to each imaging session with Qdots to label the vasculature. During the bone healing phase, at day 7, 14 and 21 post-surgery, a strong reshaping of the stromal network becomes evident from 3D fluorescence imaging in the same individual. The changes continue also at day 60 post-surgery under homeostatic conditions. Imaging volume 150x150x66 μm^3.

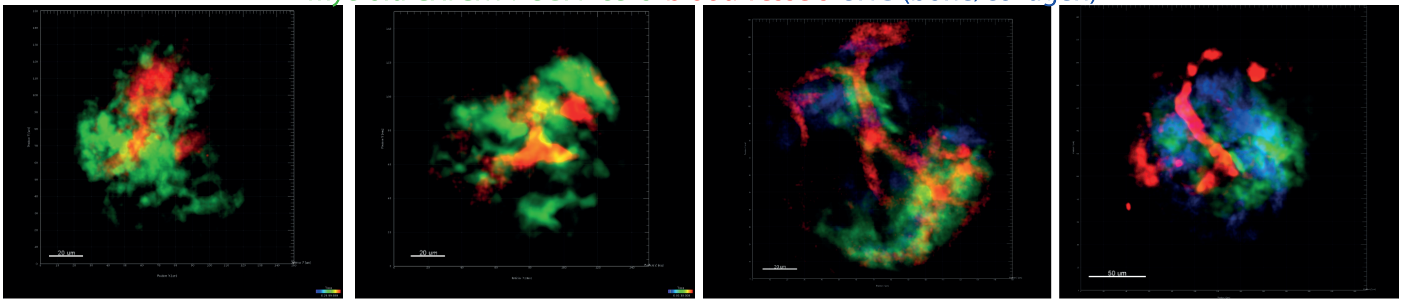

Supplementary Figure 10 | Repeated LIMB imaging using a short (500 µm long) endoscope tubing in a CX3CR1 eGFP mouse. 3D reconstructions of fluorescence images at day 3, 7, 21 and 31 post-surgery are depicted. Myeloid CX3CR1+ cells are depicted in green, blood vessels labeled by Qdots in red and second harmonic generation (SHG) originating from collagen and bone in blue. This time course reveal the initial inflammatory phase, followed by vascular remodeling and finally by soft tissue and bone growth, after drill hole injury. Hence, after 31 days we observe in front of the LIMB microendoscope a situation similar to that expected in endosteal regions.
