# Supplementary material for: Longitudinal intravital imaging of the femoral bone marrow reveals plasticity within marrow vasculature
Source: Nat Commun. 2017 Dec 18;8:2153. doi: 10.1038/s41467-017-01538-9 (PMC5735140; doi:10.1038/s41467-017-01538-9)
Supplement: Supplementary file 3 — Description of Additional Supplementary Files [file 41467_2017_1538_MOESM3_ESM.pdf]

Description of Additional Supplementary Files

File Name: Supplementary Movie 1
Description: 3D ex vivo $_{\mu}$CT of the right femur directly after implantation. The implants were fixed to the femur by two bicortical screws in an elevated position, thus they do not compress the periosteal surface of the bone. The endoscope tubing reaches the center of the marrow cavity in the distal part of the bone. Titanium alloy streak artifacts appear around the implanted parts. In the 3D-radiograph, the implant was reconstructed in Imaris for better visualization. Voxel size = 10.5 µm. Scanning in cone beam mode with 70 kVp. Scale bar = 2 mm.

File Name: Supplementary Movie 2
Description: Open field behavior of animals that underwent LIMB-implantation. The activity of mice at different time points shows no impairment in motility after surgery of the right hind limb. The same individual mouse is shown from above the cage in movie sequences at day 2, 6, 9, 13 and 21 post-surgery.

File Name: Supplementary Movie 3
Description: Intravital imaging and tracking of B lymphocytes for motility analysis in the femoral bone marrow at day 90 after LIMB-implantation. Intravital imaging was performed in *CD19:tdRFP* mice at day 90 after implantation. The mice received an intravenous injection of Qdots before imaging. Left and right: B lymphocytes are shown in green, vasculature in red. Right: Tracking the motility of the B cells revealed that some of the small (<500 µm^3) RFP$^+$ cells are motile (cyan objects/ tracks), whereas all of the large (>500 µm^3) RFP$^+$ cells are stationary (yellow objects/ tracks). Scale bars = 20 µm.

File Name: Supplementary Movie 4
Description: Intravital imaging and tracking of B lymphocytes for motility analysis in the calvarial bone marrow. Intravital imaging of B lymphocytes in the calvarial bone marrow of a *CD19:tdRFP* mouse was performed under steady state conditions. The mouse received an intravenous injection of Qdots before imaging. Left: B cells are shown in green, vasculature in red. Right: Tracking the motility of the B cells reveals that some of the small (<500 µm^3) RFP$^+$ cells are motile (cyan objects/ tracks), whereas all of the large (>500 µm^3) RFP$^+$ cells are stationary (yellow objects). Scale bars = 20 µm.

File Name: Supplementary Movie 5
Description: Intravital imaging and tracking of B *lymphocytes* for motility analysis in the femoral bone marrow at day 60 after LIMB-implantation. Intravital imaging was performed in *CD19:tdRFP* mice at day 60 after implantation. The mouse received an intravenous injection of Qdots before imaging. Left and right: B lymphocytes are shown in green, vasculature in red. Right: Tracking the motility of the B cells reveals that some of the small (<500 µm^3) RFP$^+$ cells are motile (cyan objects/ tracks), whereas almost all of the large (>500

µm³) RFP^{+} cells are stationary (yellow objects/ tracks). Small RFP^{+} cells can be observed as they enter and leave the bone marrow via the bloodstream. A single large RFP^{+} cell moves back and forth along a vessel. Scale bars = 20 µm. Grid size = 10 µm.

File Name: Supplementary Movie 6
Description: Longitudinal intravital imaging of B lymphocytes in the femoral bone marrow at day 30 after limb-implantation. LIMB was performed a CD19:tdRFP mouse at day 30 (see also Supplementary Movie 7) after implantation. The singlet GRIN lens, which gives a larger field of view of 280 µm in diameter (as compared to 150 µm with the triplet GRIN lens, compare also Supplementary Movie 3, 5) stayed stably glued within the implant during the experiment. The mouse received an intravenous injection of Qdots before each imaging session. B cells are shown in green, vasculature/Qdots in red. Vessels are well delimited and RFP- cells appear as dark shadows in the blood vessels. Video is exemplary for n = 3 experiments. Scale bar = 50 µm. Grid size = 20 µm.

File Name: Supplementary Movie 7
Description: Longitudinal intravital imaging of B lymphocytes in the femoral bone marrow at day 38 after LIMB-implantation. LIMB was again performed in the same *CD19:tdRFP* mouse 8 days later at day 38 after implantation (see also Supplementary Movie 6). For both time points, the singlet GRIN lens stably glued into the tubing was used, which gives a larger field-of-view of 280 µm in diameter as compared to 150 µm with the non-fixed triplet GRIN lens (compare also Supplementary Movie 3 and 5). The mouse received an intravenous injection of Qdots before imaging. B cells are shown in green, vasculature/Qdots in red. Number of RFP^{+} cells is slightly decreased as compared to day 30. RFP^{-} cells appear as dark shadows in the blood vessels. Video is exemplary for n = 3 experiments. Scale bar = 50 µm. Grid size = 20 µm.

File Name: Supplementary Movie 8
Description: Intravital imaging and tracking of B lymphocytes for motility analysis in the calvarial bone marrow. As an example for recording B lymphocyte migration in BM cavities of flat bones, intravital imaging was performed in the calvarium of a *CD19:tdRFP* mouse that received an intravenous injection of Qdots before imaging. Left: blood vessels/Qdots are shown in red, B lymphocytes in green, bone/collagen detected by second harmonic generation in blue. Right: surfaces of single cells were digitally reconstructed and tracked over time. Color-coding shows cells >500 µm³ and its tracks in yellow and cells <500 µm³ and its tracks in cyan. B lymphocytes <500 µm³ have significantly higher displacement rates than B lymphocytes >500 µm³ (see also Fig. 4). Scale bar = 100 µm. Grid size = 50 µm.

File Name: Supplementary Movie 9
Description: Intravital imaging and tracking of B lymphocytes for motility analysis in the tibial bone marrow. As an example for imaging of B lymphocyte migration in long bones which was recorded using a method other than LIMB, intravital imaging was performed after removing the cortex in the tibia of *CD19:tdRFP* mice. The animals had received an

intravenous injection of Qdots before imaging. Left: blood vessels are shown in red, B lymphocytes in green, bone/collagen detected by second harmonics generation in blue. Right: surfaces of single cells were digitally reconstructed and tracked over time. Color-coding shows cells >500 μm^3 and its tracks in yellow and cells <500 μm^3 and its tracks in cyan. Most of the tracked cells are sessile, lymphocytes showing high motility are exclusively <500 μm^3. Scale bar = 100 µm. Grid size = 50 µm.

File Name: Supplementary Movie 10
Description: Intravital imaging of migrating and sessile cells 2h after activation of paGFP in the femoral bone marrow using the LIMB-implant. 2 h after photoactivation of paGFP in all cells in a volume of 100x100x9 μm^3 (green) a population of motile cells is observed in the bone marrow. Over the time course of the recording (86 min., as shown in upper left corner), several cells migrate out of the activated volume. The event of one motile cell entering the blood vessel system (red) is highlighted (circle). Several other cells are observed moving along the vessel walls. Vessels were visualized by intravenous injection of Qdots before imaging. Scale bar = 50 µm. Grid size = 20 µm.

File Name: Supplementary Movie 11
Description: Long-term intravital imaging of migrating and sessile cells directly after activation of paGFP in the femoral bone marrow using the LIMB-implant. Directly after photoactivation of all cells in a volume of 100x100x9 μm^3, the majority of the cells are stationary. After 50-60 min the first cells leaving the photoactivated volume can be observed. After 90 min of intravital imaging, many migrating cells within and outside the activated volume become visible. No signs of photobleaching of activated paGFP are observed after more than 200 min of imaging. Elapsed time is shown in minutes in the lower right corner of the movie. Scale bar = 50 µm. Grid size = 20 µm.

File Name: Supplementary Movie 12
Description: Intravital imaging of vasculature and SHG in the bone marrow 115 days after surgery. 115 days after implantation of the LIMB-implant vasculature (red) and newly formed collagen structures (SHG, blue) are detected in the imaging volume. Non-fluorescent cells can be observed in the vessels as dark spots. Vessels were visualized by intravenous injection of Qdots before imaging. Elapsed time is shown in minutes in the lower right corner of the movie. Scale bar = 20 µm. Grid size = 20 µm.

File Name: Supplementary Movie 13
Description: Intravital imaging of vasculature in the bone marrow at day 35 after surgery and 24 hours later. The left panel depicts the 3D reconstruction of the bone marrow vasculature 35 days after implantation (green, 0 h and red, 24 hours later). Vessels were visualized by intravenous injection of Qdots before imaging.The right panel depicts the 3D reconstruction of the differential image of the vasculature acquired by LIMB, at 0 and 24 hours, i.e. 24 h – 0 h. Positive values, i.e. appearance of blood vessels, are depicted in cyan and negative values, i.e. disappearance of blood vessels, are depicted in yellow.
